# How do Muslim community members perceive Covid-19 risk reduction recommendations - a UK qualitative study?

**DOI:** 10.1186/s12889-021-10506-4

**Published:** 2021-03-05

**Authors:** Shaima M. Hassan, Adele Ring, Naheed Tahir, Mark Gabbay

**Affiliations:** 1 Institute of Population Health Sciences. Department of Primary Care and Mental Health, University of Liverpool, Waterhouse Building, Brownlow Street, Liverpool, L69 3GL England; 2 NIHR Applied Research Collaboration NWC, Liverpool, England

**Keywords:** Muslim community, COVID-19, Risk, Health behaviours, Communication

## Abstract

**Introduction:**

The evidence is now unequivocal that people from Black and Minority Ethnic Backgrounds (BAME) living in the UK are disproportionately affected by covid-19. There is growing evidence that the reasons for this difference are multi-factorial and need further exploration.

**Aim:**

The aim of this study was to understand better, perceptions of risk and responses to covid-19 of members of the Muslim community living in the North West of England, and to understand the facilitators and barriers to adherence to restrictions and guidance measures.

**Method:**

A total of 47 participants took part in 25 in-depth qualitative interviews and four focus groups (n=22) that explored perceptions of risk and responses to risk from covid-19. Data were analysed thematically.

**Findings:**

Participants were aware of the mechanism of transmission of covid-19 and took steps to mitigate risk of transmission including, observing a range of hygiene practices and following social distancing guidance. Increased risk of covid-19 for BAME populations was explained largely in terms of exposure to the virus due to the types of employment people from BAME populations are employed in. Limitations both within the working environment and more generally in public spaces, was identified as problematic for effective social distancing. The closure of mosques sent out a strong message about the seriousness of the virus and religious teachings reinforced hygiene and social distancing guidelines.

**Conclusion:**

Across society there are people that adhere to restrictions and guidelines and those that do not. Improving local information provision and communication pathways during times of the pandemic, could aid understanding of risk and promote adherence to social distancing restrictions.

**Supplementary Information:**

The online version contains supplementary material available at 10.1186/s12889-021-10506-4.

## Introduction

The covid-19 pandemic is linked to many deaths across the UK and worldwide. Growing evidence shows that covid-19 does not affect all members of the population equally, with those living in urban areas and in more deprived areas being disproportionately affected [1]. Evidence also shows that people from Black, Asian and Minority Ethnic (BAME) backgrounds are being harder hit [1, 2]. In the UK ~ 19% of hospital deaths have been among BAME groups and people from BAME groups account for 35% of critical care unit patients in England [3, 4]. These differences in mortality from covid-19 between groups in society are increasingly being explained in terms of differences in demographic, geographic and socio-economic factors [5]. These highlight stark and continuing health inequalities experienced by disadvantaged [6] and specifically BAME populations [4, 6]. BAME groups often experience poorer socioeconomic circumstances including, living in more deprived neighbourhoods with overcrowded and multigenerational households, employment in essential occupations and reliance on public transport [7–9]. They also experience higher levels of comorbidities and differential access to appropriate and adequate healthcare services [7, 8]. These circumstances compound effects of covid-19 and its transmission across different BAME groups.

On the 23rd March 2020 the UK government introduced social control measures to slow covid-19 transmission. These included ‘stay at home’ message (with the advisement to leave home for essential reasons only), a 2 m distance to be maintained between individuals from separate households (wherever possible) and the requirement to self-isolate away from others within the household if experiencing typical symptoms of the virus [10, 11]. On the 11th of May 2020 the message changed to ‘stay alert’ with some restrictions being relaxed [12, 13]. This study took place during this initial lockdown period that commenced on the 23rd March during the ‘stay at home’ period of restrictions and continued on through the subsequent more relaxed ‘stay alert’ restrictions imposed on the 11th of May, but was completed before the reintroduction of restrictions locally or nationally as infection rates rose later in the year.

The capacity of populations to protect their health and adhere to hygiene, social distancing and self-isolation measures, is likely to reflect current health divides and the adequacy of social and welfare systems and their environment at work and home. This differential capacity within populations is also a likely factor in the differential mortality rates between populations and areas. Compliance and response to local lockdown measures may vary across population groups; with people from different ethnic backgrounds varying in behaviours, comorbidities and immune profiles, and consequently their risk of infection and its consequences [7]. The Muslim population makes up the second largest religious group in the UK [2]. There are growing concerns that the Muslim community in the UK are particularly vulnerable to covid-19 [1, 2, 14]. Reports indicate that the highest age-standardised mortality rates (ASMRs) of deaths involving covid-19 were within the Muslim group, with 198.9 deaths per 100,000 males and 98.2 deaths per 100,000 females within the period 2-March to 15-May 2020 [15]. The Muslim community in the UK often come from multi-generational households, have comorbidities and a relatively higher proportion live in areas of deprivation [1, 2]. Such conditions can increase the risk of covid-19 infection and create barriers to social distancing or effective self-isolation.

Muslims living in the UK are not a single homogenous group [3], however, Muslims generally will respect the recommendations by Islamic teaching to maintain family kinship, care and look after elderly family members, and pray or breakfast in congregation [16, 17]. There are also other traditions that some Muslim groups hold including, eating together (often from one plate) and sharing utensils, or intimate social behaviours such as kissing the hands or the heads of elderly family members [16.17]. These practices, make the current covid-19 interventions of social distancing and self-isolation ‘alien and absurd’ to Muslims [14, 16, 17]. Health officials have highlighted ‘Muslims as being one of the at greater risk populations/groups because of a number of cultural factors’ [14]. There have been reports that point the finger at Muslims for the increase spread of covid-19 within the community [18–20]. However, this can be seen as ‘othering’ and minority/victim blaming [21]. The need to untangle these different factors and understand better how people are responding to infection control messages has been emphasized [7].

The aim of our study was to explore the perceptions of risk and experiences of social distancing and social-isolation recommendations to reduce the risk of covid-19 transmission, of Muslims living in the North West of England. This study is part of a wider COVID-LIV Area-B study (incorporating a number of qualitative social science sub-studies that are part of the larger COVID-Liv household virology research) exploring perceptions of risk and experiences of the covid-19 pandemic for households, communities and organisations in one region of the North West of England.

## Method

This study used qualitative in-depth semi-structured interviews (*n* = 25) and four focus group (*n* = 22) with members of the Muslim community living within the North West of England. The strength of this approach is that it enables the researcher to explore in depth, the phenomena that are under investigation. This approach has particular utility when exploring previously under-researched areas since through the generation of in-depth data, a more thorough understanding of the phenomena under study can be gained [22]. Though the knowledge gained through this research approach is not generalizable in the same way as quantitative research findings, it provides richer and more nuanced accounts of local phenomena that cannot be known or understood through quantitative methods of investigation.

### Participants and recruitment

Through purposive sampling, people from the Muslim community living within the North West of England, aged over 18 years and who were English speakers were invited to take part in the study. Potential participants were invited (via email and social media which included study information and researchers contact details) via established Muslim community groups including, Local Muslim mosque groups (via WhatsApp groups, Facebook and email), and other local Muslim groups/networks identified by a public adviser [NT] working with the lead researcher [SH]. Both NT and SH come from a Muslim background.

Potential participants who registered their interest in the study were provided with an electronic copy of the study information sheet and consent form. They were followed up with a phone/zoom call the next day or at a time convenient for them, to ensure that the study’s details are discussed and what would be involved should they agree to participate.

Potential participants that agreed to take part in an interview were given the option to do so via telephone or online video call (Zoom) as appropriate for the participant. As for the focus groups, potential participants that agreed to take part in a focus group were invited to join one of four scheduled online focus groups meetings on Zoom.

Interviews and focus groups were audio recorded with participants’ permission with interviews lasting between 30 to 45 min and focus groups between 60 to 75 min. Prior to interviews and focus groups, participants were asked to read and sign the study consent form (providing an electronic signature or typing their name into the consent form, or signing and scanning the form) and returning it to the research team via email. At the start of each interview and focus group, consent was reviewed and verbal consent was also recorded. Audio recordings were transcribed verbatim by a professional transcriber and transcripts were reviewed for accuracy and anonymised by members of the research team prior to importing into NVivo software (qualitative data analysis software) to support the management and coding of data.

### Data collection

Data were collected through one-to-one interviews and focus groups. This approach is called triangulation, which is the use of several research methods to explore a research question in order to enhance the robustness of data and ensure findings are comprehensive and well-developed [23]. The use of these two independent data collection methods in combination provides a more comprehensive understanding by expanding the breadth and/or depth of the findings [24]. For example, one-to-one interviews provides a deeper insight to explore personal experiences, whereas focus groups interactions emphasize groups’ similarities and differences and give rich information about the range of perspectives and experiences to examine opinions and beliefs about the study’s phenomenon [24]. This approach helped facilitate a deeper understanding of Muslim community experiences of covid-19 recommended measures.

Three interviews were conducted via Zoom (online video call) and the remaining 22 interviews were by telephone, and four focus groups were conducted via Zoom (video call) with a total of 22 participants. An interview guide was developed by the research team [SH, AR, NT and MG] and included semi-structured questions with prompts to facilitate discussion (Additional file 1). The topic guide promoted questions like ‘what religious and/or cultural practice that could have influenced your response to covid-19’.

Interviews and focus groups were carried out during the initial lockdown period that commenced on the 23rd March during the ‘stay at home’ period of restrictions and data collection ended in the first week of July 2020.

### Data analysis

Using thematic analysis as a framework to interpret the study’s findings, members of the research team [SH, AR, NT and MG] initially blind coded transcripts and then worked together to identify codes and create overarching main themes. All researchers discuss the identified codes and identifying themes informed by symbolic interactionism [25] and social constructionism [26], sought to elucidate the meanings participants ascribed to covid-19 virus and health protection recommendations (social distancing and social isolation). We also sought to understand how these meanings were constructed, by paying attention to participants’ interactions with the world around them and the people and objects within it and their interpretation of these, in the context of the pandemic.

Using this framework the research team read through interview transcripts and started to organise the data into potential themes. The initial themes identified were explored further with focus groups data and any new themes were added to the developing framework. The initial themes from both data sets were then revisited as a whole and main overarching themes and sub-themes identified.

### Public involvement

ARC NWC Public Adviser [NT], as a member of the study team, reviewed study design and materials, supported initial contact and link to the Muslim population, undertook data collection and analysis, and contributed to the writing and review of this paper. The research team [SH, AR, NT and MG] and additional Public Advisers, reviewed the themes presented in this paper.

### Ethical approval

This study received University of Liverpool ethical committee approval [Ref:7685].

## Results

The Muslim community are not homogenous group in their nature but they are community within community, with different ethnicities, languages, backgrounds, cultures and traditions. Therefore, the study sought to widen the recruitment of participants to provide a representation of diverse groups among Muslims with the North West.

A total of 47 participants took part in the study, 25 were recruited to take part in individual interviews (see Table 1) and a separate sample of 22 participants were recruited to take part in one of four focus groups.

**Table 1:** Interview sample characteristics

Participant	^**a**^Age/gender	Participant identified themselves as a key worker	Participant identified other member of household as key worker
P1	Middle-aged female	No	No
P2	Middle-aged female	Yes	No
P3	Younger-aged female	No	No
P4	Younger-aged female	No	Yes
P5	No age given	Yes	Yes
P6	Younger-aged female	Yes	Yes
P7	Younger-aged female	No	No
P8	Middle-aged female	Yes	No
P9	Middle-aged female	No	Yes
P10	Younger-aged male	Yes	No
P11	Younger-aged female	No	No
P12	Middle-aged male	Yes	Yes
P13	Middle-aged female	No	Yes
P14	Younger-aged male	No	No
P15	Middle-aged female	No	Yes
P16	Late middle -aged male	No	Yes
P17	Younger-aged male	No	Yes
P18	Younger-aged male	No	Yes
P19	Middle-aged female	No	Yes
P20	Younger-aged male	No	Yes
P21	Middle-aged male	Yes	Yes
P22	Late middle -aged male	No	No
P23	Younger –aged male	No	Yes
P24	Late middle -aged male	No	No
P25	Late middle -aged male	Yes	No

^a^Younger age adults (19–34 years); ^a^Middle-aged adults (35–49 years); ^a^Late middle-aged adults (50–65 years)

A total of 25 participants (12 Males and 13 females) were recruited to take part in individual interviews. Their ages ranged from 19 to 65 years (Mean: 37.5), for those who provided age. The number of people living within participants’ households ranged from 2 to 9 with the age range of household members being from 11 months to 65 years. The majority of participants (17) were in paid employment, five of whom were self-employed. Three participants were not currently working in paid employment, and five participants were in higher education. The majority of households (18) had at least one key worker living in the household.

### Focus groups sample characteristics

A total of 22 participants joined the four focus groups. Specific participant demographics were not collected due to the potential of too much personal disclosure would influence the focus group dynamic. Across the four focus groups 10 males and 12 females with ages ranging from 18 to 60 years and representing a range of occupations and employment status (employed, unemployed and higher education) took part.

Participants’ ethnicity in both interviews and focus groups as defined by themselves included: British Arab/British Yemeni/Arab, British Pakistani, Indian/Asian/Bangladeshi/Pakistani, White European, Black British, Somali, and Black African.

The study sought to actively involve members of the Muslim community across the age range including those over the age of 65 years. However, in spite of seeking to purposively sample older members of the community we were unable to recruit any participants over the age of 65. As such, we have been unable to explore the views of this older age group directly, although some participants discussed views held by older members of their family and wider community.

#### Qualitative findings

The meanings that participants ascribed to the virus and imposed restrictions were constructed through participants’ interactions with the world around them including other people, objects and information and their interpretation of these.

We identified six themes that helped us to gain a better understanding of participants’ perceptions of risk and their experiences of and views about imposed restrictions: 1) Reality and seriousness of the virus; (2) Exposure; 3) Beliefs and behaviour – taking precautions; 4) Challenges to adherence and self-protection; 5) Conflicting messages and information and 6) Faith and adherence.

#### ‘Reality’ and the seriousness of the virus

Perceptions of risk were varied and highly context dependent, with participants’ assessment of risk for themselves, their household and the wider community, firmly embedded within their day to day lives, their roles at home, at work and within their wider community and their individual health status (e.g. being categorised as ‘vulnerable’).

I think, from my point of view, there is a risk as my husband is a key worker, he’s a doctor … so he can bring that virus at home he can give it to me or I can pass it to kids, to my community if I not isolate myself if I’ve got the symptoms (P9).

Participants’ understandings of covid-19 included, their perception about the *seriousness* of the virus, perceptions about risk of contracting the virus and their beliefs about how the virus was transmitted. Participant’s beliefs about the *seriousness* of the virus were influenced by their lived experience of the virus which changed overtime. For example, one participant described how people’s interpretations of the virus as something to be taken ‘seriously’ changed as the virus got *‘closer to home’*.

It was something in China … but it wasn’t near us I think only in the UK it was only taken seriously then around that time [beginning of March] … but before that people didn’t like say ‘oh it’s maybe just a flu’ (FG3).

For some participants it wasn’t until they actually contracted the virus or a close family member became seriously ill with the virus that the virus became ‘real’ for them and they started to take it seriously.

Before my parents got it I almost thought of it as very like a mystery or something which is very intangible you don’t know what it is. But when they got it and obviously my mum went to hospital as well and she was admitted for a couple of days and it was quite serious for while it then it became quite serious for me as well (P4).

Another participant described how her father failed to take the virus seriously until the family became ill. This participant also highlighted how other cues, for example, a letter informing her father about his vulnerable status, might have influenced his thinking about the seriousness of the virus, had it come sooner.

If we received the information to say that he [dad] is in vulnerable category early on that would have allowed my dad to take seriously. Because before we get quite ill it was a joke to him, it wasn’t a thing. I think it was really difficult for him to get his head around it and I don’t really blame him because we had a lot of questions ourselves (P2).

The closure of the mosques was another important cue or sign as to the seriousness of the virus, identified as important by participants because the mosques are central to community worship that is a key part of Muslim community life and consequently mosques are rarely if ever closed.

I said closing Mosques down was a big message for community that we are experiencing serious disease yeah so you know Mosque will never be closed just if not this, coronavirus (FG1).

Mosque is something we hold close to ourselves as Muslims and for it to be considered so serious that we can’t even go to the Mosque, it would have opened a lot of people’s eyes to the seriousness of the situation (P18).

Participants’ thinking about the reality and seriousness of the virus was also influenced through various information sources. For example, some participants followed both UK and other news channels such as Al Jazeera. One participant described how the differing reports from these channels led to uncertainty about the seriousness of the virus for UK citizens.

So initially what I knew about covid-19 I think like most of us depended upon the news and I particularly rely on BBC and Al Jazeera. So through the BBC and Al Jazeera I noticed that the BBC was taking more of a conservative stance. So it was sort of reflecting what the Government was telling us and the, we knew that Covid was spreading quite rapidly in other countries. So in Italy it was serious … but the BBC sometimes didn’t reflect that and Al Jazeera was reflecting it more. So we were unsure what really the severity of coronavirus would be and its affect would be on in British citizens (P20).

Another participant described how the family felt the need to convey the importance of the virus to their mother because she wasn’t accessing the news and they were unsure as to whether her usual sources of information (friends) were *‘acknowledging the seriousness’* of the virus.

But my mother doesn’t get her information from the news, my mother gets her information from her friends and from family and so we’ve been quite keen that, I don’t know the conversations she’s having with her friends and I don’t know whether they are acknowledging the seriousness of this. So I have to make sure that myself and my sisters for example when we speak to our mother we have to get across the seriousness of this [virus] (P16).

Participants’ experiential knowledge gained for some through their working roles (in particular those in the medical profession) also influence understanding about the seriousness of the virus. For others, their access to more medicalised information was via family members or friends.

Alhamdulillah I’ve been I think it’s my job that’s kind of like grounded me. Perhaps and this is the truth perhaps if I’d worked in a different field maybe I wouldn’t have been as firm and stricter as I am now (P1).

#### Exposure

A key theme in participants explanations of risk were that of ‘*exposure’* to the virus - considered more likely for those in key worker roles and for those involved in key household task (e.g. shopping), where contact with others who might by carrying the virus or with objects that they might touch and become infected through, was more likely.

My family risk, like my son working as well as my daughter and my wife at home. My wife working from my home. So I’m not working at the moment. So if one person get it like this corona, all family get very big risk (FG2).

Exposure through limitations of (PPE) early on in the pandemic was also a concern. This coupled with ‘inadequate’ guidance, left some participants feeling ‘unsafe’ and unable to protect themselves or their patients from risk of exposure to the virus.

I feel it’s [guidelines] highly inadequate, it’s not safe for the staff it’s not safe for the patients and on top of that there’s no we don’t have the resources, we don’t have adequate PPE and we don’t have separate, cross transmission is very easy. Either from patient to staff or even from staff to patients (P8).

I know from one of the patients actually who was a doctor from ethnic minority; she told me when she started wearing the face mask she was told to take it off because she’s scaring the patients (P3).

The concept of exposure was also central to participants’ reasoning for why people from BAME groups were at greater risk.

Especially when you think of the jobs that black and ethnic minority people do, a lot of them work in the NHS, a lot of them are doctors/nurses even as far as taxi drivers or working with the public or in job roles that that mean that they’ve got to have contact with people all the time (P15).

I think there’s socioeconomic background of a lot of BAME communities is another factor and the fact say from a Muslim perspective we are very sociable as a community or communities, whether you’re sort of Arab, Asian, African you tend to have backgrounds of living if not with family having a lot of involvement with your family even day to day interactions … so I think there’s lots of that physical contact is very much part of it hugging and shaking hands and so I think there’s a combination of things that probably makes us more at risk (P16).

Several participants described how they felt that people from BAME communities (particularly those working within health care settings) could be at greater risk of being exposed to the virus because of *‘institutionalised racism’*.

Especially certain grades of doctors for example middle grades, they tend to work in places like [other city] which was hit really badly or [other city] where not necessarily other doctors would, which I had read a bit and it’s very difficult because you just don’t know but whether there is a little bit of sort of institutionalised racism in the NHS (FG4).

[BAME] they found themselves leading teams of the corona wards …. I mean none of them are white British and they found it bizarre that suddenly they found themselves doing ward rounds on a coronavirus ward when the fact that it’s not appropriate, because that’s not their specialty or they feel like unequipped to do it … they’ve been pushed to the front to do it. That’s from staff perspective. I know like one white English consultant, I haven’t seen him since January … .and all you see is the names on documentation and the title is remote review (P21).

One participant described how they were asked to complete a risk assessment because they were from a BAME background, however, their perception was that this meant little in real terms and was essentially *‘a tick box exercise’*.

The whole thing (risk assessment) took like a minute and a half and I just went straight back onto the ward, I spoke to the ward where most people are some kind of ethnicity and we were all discussing and were like ‘yeah it’s a tick box exercise’ (FG3).

#### Challenges to adherence and self-protection

Although participants considered social distancing and isolation measures to be important in helping to reduce the risk of virus transmission, their experiences highlighted some key challenges for themselves and others in adhering to them.

### Assessing risk based on symptoms

Determining whether symptoms were those of covid-19 was not straightforward. A cough was considered an ambiguous symptom in the absence of other covid-related symptoms and a lack of testing in the early phases of the lockdown. This ambiguity could lead some to ‘work through’ symptoms.

I know a few people who have had the corona and at first they just thought it was nothing, it didn’t show, they didn’t have the temperature but they had like sort of a cough or whatever but they worked through that because it’s just that ‘oh I need to work through that and just carry on’ but it was only until they’d get tested and then they go ‘oh yes I’ve got it’ but you would have never thought (P5)

### Economic need

Some participants spoke of the tension between adhering to self-isolation recommendations in the context of economic need. Low wages and ineligibility for financial support, self-employment, responsibility for the livelihood of employee’s and the issue of the additional cost for private businesses of paying someone to come in and cover their work whilst they were self-isolating, were all identified concerns and potential barriers to self-isolation.

A lot of BMEs perhaps come from poverty, perhaps come from not the United Kingdom, therefore the financial incentive is a big thing and they need to be out there in order to feed their loved ones and putting themselves at risk (P1).

I mean one of the guys he was a taxi driver and he actually contracted covid-19 because he had to go out and he died actually contracted it because he had to go out on the taxi. The reason why he went there when I speak to his family is because they had no food, they had nobody that he can talk to get some food (P12).

### Structural limitations of the environment

Structural limitations within working and home environments could make 2 m social distancing impractical. Whilst not explicitly stated, the sense that participants were being exposed to the virus due to such limitations within local community settings, was intimated.

Their shops don’t have the right infrastructure again when you’ve got a company of [supermarket] size or a company of [supermarket] size it’s easy for them to implement it because they’ve got the financial capability to do it. But when you’re a local shop and your shops already small enough and you’ve crammed everything in there (P23).

Other aspects of working environments also made 2 m social distancing impractical, including computer stations that were fixed and social spaces such as common rooms with limited space to adapt to meet social distancing requirements.

There are processes that you’re supposed to social distance but you can’t physically, you are literally sat shoulder to shoulder next to a computer if you need to access a computer or when you’re having lunch at the table, the environment doesn’t help (P21).

Many participants questioned whether self-isolation was possible, due to constraints of home environments. For example, one participant described how limitations of space in the context of being a mum to very young children, would make self-isolation impossible.

Oh my God I don't know that [self-isolation] was a very big worry for me because I have little one, he still sleeps next to me … my house is small, there is no extra like room somewhere I could go actually, I don't know what I would do, I think it’s be everyone will get sick because there is no other way for me, nowhere to go or escape. (P11)

### Perceptions of self – cultural challenges

Participants also spoke of the challenge of adhering to social distancing recommendation for their community where sense of self was so deeply entrenched within the togetherness of the community in both a spiritual but also physical sense.

It’s the norm to be around people to have people over, for you to go over to theirs to see them in the shop and to give them a hug or shake their hand and I can imagine that in this situation people still want to do that because it’s just part of the culture. It’s just still part of like who they are and what they are as a community and because it’s kind of ingrained in them they might feel like a bit bad if they don’t just continue to be like that … and because of that there’s still that kind of spread of infection which is higher than non-ethnic minority communities (P7).

The fact that lockdown took place during the month of Ramadan and the celebration of Eid where members of the Muslim community would usually break the fast in congregation, was a particular challenge, with one participant reporting that whilst they had stayed at home they had heard of other households meeting up.

I definitely heard of people meeting up on Eid … like different households so that’s obviously up to them. Personally I didn’t I didn’t meet up with anyone (P17).

#### Conflicting messages and information

There were many references to the inadequacy of information and guidance in the early stages of the pandemic. There was no guidance for people running small independent businesses, leaving them to look to their neighbours for guidance as to how to respond and relying on their own ‘common sense’.

The smaller shops there’s, no one telling him you have to do this … I mean if this [covid] wasn’t on anyway he would [husband] have the health inspector in there every quarter of the year do every 3 or 4 months they come in … so why can’t they come in now and say this what you need to do (P5).

It was felt that guidance could have been clearer and that the constant changing of information (often on a daily basis) and increasingly so as the pandemic went on, was a cause of uncertainty as to how to best respond.

Conflicting information again … the stay alert thing from Boris, someone mentioned it before absolute nonsense I mean not necessarily wrong, but the confusion he put out there when people were waiting for a message from … we were like nobody knows how to proceed from here now … all the clear directions are all sort of gone, looking at each other like so what does this mean for us (FG3).

### The behaviour of others

Participants’ narratives highlighted the importance of the behaviour of other people in conveying messages about the virus to the community and beliefs about the seriousness of the virus had implications for adherence to social distancing measures.

I don’t think that (staying indoors, not going out unless necessary) has been implemented throughout the society, what we hear on TV advice and so it’s not really practiced in reality … we go to shops there’s no 2 m distance, there’s no in the street no 2 m distance and depending on locations of the city there’s not, (P24).

For some participants the non-adherence of others, particularly those in positions of authority, sent mixed messages to the wider community about the need to observe social distancing measures.

I don't know if it’s because we’re seeing on the news as well so many like government people not really listening and things so it makes you think oh maybe we can’t, we shouldn’t listen or, I don't know. But I've seen that that people now, I feel that people if they're not necessarily listening to the guidance it’s just a case of you do what you want to do (FG4)

There was also the suggestion that early messages did little to convince people about the seriousness of the virus and the need to adapt their behaviour.

Very early on when we were all hearing a lot of young people are fine it’s the older people that are being affected as if that was ok and we were supposed to be ok with older people being at risk. I was like ok I understand what you're trying to say and like it’s not necessarily like the plaque that’s wiping everyone out, but there's still a significant risk and people were just kind of like ‘oh well if it’s not affecting me and my friends I don't really have to change the way I am’ (FG3)

### Barriers to local communication

The closure of mosques although strongly supported, also meant that usual channels of communication (through respected leaders) were absent during lockdown. This, coupled with a lack of information in languages other than English, meant that key messages may have been missed by some members of the Muslim community.

So we should have this type of people who can lead us and who can guide us … they should take; they can do a lot of thing like Mosque head or like community head …. I didn’t hear anything but I think we should have (FG4).

Participants advocated for more localised guidance with recommendations delivered through respected leaders within the community and better communication between national government/local government and community leaders to develop culturally appropriate guidance.

I think possibly need to communicate with the religious leaders within those areas, especially within Asian communities … there sometimes more reliance upon religious leaders and they’re seen a voice of reason and if we were able to communicate with them and then they can communicate with the people within the community that they have contact with … then it can enable sort of government advice to be easily disseminated as well (P20).

#### Beliefs and behaviour – taking precautions

Participants described how the virus was *‘easily’* spread and transmitted through droplets that could be inhaled through the air and land on surfaces where it could be pass on through touch. These beliefs reinforced the need for hygiene practices and social distancing.

I was quite worried for my daughter actually when she came so I made her wear the Hebei and gloves just in case and anything she touched was contaminated (P3).

Participants also described additional precautions they were taking to reduce risk of transmission, including wearing face masks/covering (well before this became mandatory), wearing gloves, using hand sanitisers and disinfecting food packaging before putting it away.

Even when my husband goes [shopping] he wears a face mask and he takes the hand sanitiser, when he comes home we have this antibacterial wipes, I wipe everything all the shopping, whatever comes in my house we clean it thoroughly or leave it for a few days, they said like it will die the virus without the body (P11).

Participants working in healthcare environments described the particular routine they carried out on arriving home from work and prior to greeting other household members, to try and limit risk of transmission.

I’m very scared. We come home our routine is when we come home we get changed by the door, we all come in separately … we’ll come in strip our clothes off, put it in a carrier bag … take that to the washing machine then straight up shower … that’s not been told by anyone by the way … this is what we’ve done ourselves (P5).

Participants’ descriptions of their actions to reduce risk suggested that many of the usual social and cultural practices whilst very hard to relinquish, were actively being discouraged within the community with alternate forms of social interaction becoming more acceptable.

As Muslims we shake hands and give hugs to each other and this it’s our own nature … .we can’t just give it up just easily and this is a big challenge for all of us as Muslims. We want to protect ourselves as a community and protect the others of course I can see a lot of Muslims just following these measures (FG1)

#### Faith and adherence

Participants’ faith was an important influence on their beliefs and consequently their actions to minimise risk for themselves and others. Key religious text that offered direction regarding action were important in supporting directives to socially distance and self-isolate where necessary.

In terms of the risk actually Islam mandates you to take necessary precautions and at the time of the Prophet and Companions when they were plagues they would take precaution and they make sure people in there so essentially practiced isolation (P10).

Religious text were important in the context of the closure of the mosques and disruption of usual religious practices such as breaking fast in congregation at the end of Ramadan. Participants described how they interpreted these changes with reference to their faith and in so doing, did not feel they were making unacceptable compromises, which might have influenced their adherence.

The correct understanding is that this situation allows you to pray at home and you will still be acceptable and permissible and you are not meant to risk yourself for any such situations (P4).

I think it’s a good idea [closure of mosques] because it’s one of the main places for gathering so it [virus] will be transferred. It will be if anybody’s carrying it then it will definitely go to a lot of people and that’s part of the where the gathering is not allowed. (P25).

### The practice of cleanliness

Religious teaching and practices were also important in reinforcing hygiene practices including frequent hand-washing with members of the Muslim community required to maintain cleanliness in preparation for regular prayers that take place throughout the day.

One thing I do really like is that the emphasis on cleanliness you know we have that within our communities anyway you know we wash 5 times a day for prayer (P15).

### A sense of responsibility (duty)

A key concept within the Muslim faith was that of responsibility - to Allah, oneself and to others, this sense of responsibility affirmed the need to follow government guidelines.

As Muslims we have a responsibility to God, ours and others lives as well and whether that means whether that’s Government guidelines that are out at the time or whether that’s just common sense then then it has to be applied (P15).

All had a duty to protect their customers, their service users as well as their worshippers in the Muslim community as well so quite positive in that sense because all of them were quite welcoming and understood that this needed to be done (P12).

### Provision of support

Being able to self-isolate or shield was also contingent on support from outside of the home. One participant spoke of early issues where systems had not been put in place to support those who were vulnerable and isolating, whilst other participants described how the community were involved in delivering essential goods to those who were in need enabling them to shield.

For example if someone is vulnerable and they can’t go out and do the shopping, how can they do it? Who can deliver it for them? Even though at the [NHS site] we had to call the head office and ask them if some of our patients are self-isolating what shall we do? … they ask for a delivery and the driver said ‘I won’t be able to take this risk’ and then they told us about the measures of knocking at the door, staying away, putting the medication on the floor. I think these things came a little bit late (P6).

## Discussion

Our study explored the perceptions of risk and experiences of covid-19 social distancing regulations of members of the Muslim Community in the North West of England. Our findings show how participants’ interpretations of the virus as *‘serious’*, their beliefs about how the virus was transmitted and their perceptions of risk of contracting the virus (for themselves and others) influenced their responses to covid-19.

Additional precautions were taken by those working in environments perceived to be placing themselves (and their household through contact with them) at greater risk of contracting the virus. Our findings in this respect contrast with a recent USA survey undertaken in the early stages of the covid-19 pandemic, with perceived consequences for others not identified as motivating of hygiene behaviours [27]. There are perhaps two likely explanations for this difference, first, in our sample, some participants perceived themselves to be at greater risk of contracting the virus because of their exposure to it through their working role. A recent study of health compliance in the context of covid-19 found that general anxiety/fear of covid-19 was predictive of social distancing and hygiene behaviours [28]. Economic need was also identified as a driving force leading to greater exposure to the virus due to low wages, self-employment in occupations where working from home was not an option and people were ineligibility for financial support. A review of covid-19 impact within BAME groups, highlighted the impact of these types of long-standing structural inequalities and the need for urgent action to address these [29].

Secondly, is the sense of responsibility that is instilled within members of the Muslim community through their faith that mandates that individuals have a duty to protect not only themselves but also others from harm. Participants described how some within their community (similarly to some individuals within the wider population) were not adhering to social distancing recommendations. Explaining why some people adhered to social distancing rules whilst others did not, is complex.

Evidence from previous studies of compliance suggest that compliance is influence by demographic, instrumental (individual as a rational, self-interested being) and normative (internalized norms of justice and obligation) factors [30]. They suggested that if compliance is influenced by instrumental factors (self-interest), then in the context of covid-19, one might expect that those at increased risk from the virus would be motivated to comply with restrictions, whilst the concept of risk would have little influence on those within lower risk groups. Our findings would seem to support such a proposition, participants in our study perceived those not adhering to social distancing rules to not be taking the virus seriously, as evidenced by their failure to maintain social distancing recommendation in public places, even when asked to do so. Murphy et al., have explored the relationship between demographic, instrumental and normative factors, and compliance with covid-19 social distancing restrictions and concluded that Australians were motivated to comply not because of concerns for themselves or others but instead, because of a sense of duty to support authorities [30]. A number of studies have reported a link between perceptions of risk and compliance with health protection behaviours during times of pandemics. A 2010 review of papers focusing on determinants of protective behaviours during pandemics [31], found that greater perceived susceptibility to disease and perceived severity of disease was associated with adoption of health behaviours during pandemic. A telephone survey in Italy exploring the relationship between a number of variables (including risk perception) and compliance with recommendations during the H1N1 pandemic also found that perceptions about the severity of illness and risk of catch the virus was associated with compliance with some recommended behaviours [32]. More recent covid-19 studies of compliance offer conflicting findings concerning the influence of perceived risk to self on health protection behaviours. A USA study, reported that feelings of personal risk of contracting covid-19 influenced people’s tendency to engage with hand-washing and social distancing measures but perceived risk of severity of the virus did not [27]. An international questionnaire study of beliefs and attitudes about covid-19, found that perceiving oneself as vulnerable to covid-19, perceived severity of catching covid-19 and trust in government were less important predictors of taking health protection precautions than the belief that taking health protection measures would be effective in avoiding covid-19 and prioritizing one’s health [33].

Humans as social beings often look to others for signs as to how to behave within social settings, with shared norms being important in promoting cooperation between members of society [34]. However, following social norms and adhering to centralized rules is contingent on recognition of laws as essential for social order and that compliance must override personal interest [29, 34]. Social expectations or norms that influence adaptive decision-making may come into conflict when certain choices are beneficial for the individual whilst societal rules mandates a different course of action [35]. In our study, provision of information and guidance was noted to be problematic for a variety of reasons, leading some to question the legitimacy of the virus and its seriousness. This point is important, given that inconsistent information from authorities can lead to questioning of information credibility and willingness to quarantine if sick [36]. Mismatches between messages centrally and the actions of members of the local community and prominent figures in government, constituted a confusing array of signs about how people should best respond to mitigate risk. Messages that highlighted minimal risk to the many whilst more severe risk to the relative few, was suggested as a sign to some, that there was no need for them to change their behaviour. Based on previous evidence, such a message would be unlikely to motivate those that perceive themselves to be at minimal risk to adhere to restrictions. Participants acknowledged that conflicting signs could lead some to question the seriousness of the virus and consequently the need to take action to mitigate risk. These types of value-based decisions are particularly tough to determine, and require a complex trade-off between self and other regarding motivations [34]. Adaptive decisions often require integration of information from several sources that are weighted according to their respective reliability [37]. A particular issue with regard to information getting to the community was the loss of usual channels of communication through the mosques. This was problematic not only in getting a message that was understood, but also one that was recognised has having authority within the community. Lack of trust within BAME communities due to mixed messages during covid-19 pandemic has recently been reported [29]. This is problematic given that trust in authorities is important for compliance with health protection behaviours during pandemics [38] and it has been noted previously that faith-based credible information sources such as Muslim Council of Britain that are trusted by local communities are important in conveying public health messages [39]. Recent evidence from a study of compliance with covid-19 measures among youth advocates leveraging trustworthy individuals in the community to disseminate Public Health messages [40].

The lack of information provided in a culturally appropriate format - including in the languages commonly used within the community, was identified in our study as problematic. Proposals for future improvements included a need for localised communication of recommendations delivered through respected leaders within the community. Better communication between national government/local government and community leaders in developing culturally appropriate guidance was also advocated, a recommendation echoed in a recent review of covid-19 impact within BAME populations. This called for government to work together with faith leaders and community groups to identify ‘effective channels’ through which to disseminate information and provide support [29].

Participants in our study believed that adhering to social distancing and social isolation recommendations was important, but also highlighted the environmental constraints, limitations of PPE and social and cultural pressures that influence people’s decisions and responses in the context of the pandemic. Structural and economic problems during times of pandemic may be more complex and costly to solve, but need to be addressed to overcome institutional and structural barriers to behaviour modifications [29].

We noted in the introduction to this work that asking people to socially distance from others within their community was at odds with normative cultural and social beliefs and values within the Muslim community. Our findings indicate that whilst adhering to social distancing rules was undoubtedly challenging and particularly so in the context of the month of Ramadan and Eid festival, including not being able to break the fast together in the usual way, most participants noted that these departures from normative practices were wholly acceptable and in line with religious teachings. Evidence suggests that such obligations, including duty to obey authoritative figures are associated with enhanced compliance to covid-19 restrictions and that sense of obligation is instilled at a young age [30, 41]. Our evidence does not indicate, contrary to prejudice claims, that Muslims ignore advice [29] and were congregating in large gatherings as a norm. This can be seen as othering and victim blaming [21], especially in social and popular media [18, 19]. These behaviours were alleged to explain spikes in BAME communities and areas of the country with higher proportion of BAME populations [20].

Finally, it is important to note that in order for people to able to socially isolate and shield in their homes, resources and support need to be available to them. Participants spoke of delivering essentials such as shopping and medications to neighbours and elderly members of the community.

### Strengths and limitations

This is one of the first studies to conduct an in-depth exploration with members of the Muslim community in the UK about their perceptions and experiences of covid-19 and the pandemic. We have been able to identify key insights regarding how information and communication can be improved in the future to ensure that members of the Muslim community have access to information about risk and actions to minimise risk. Also whilst there have been some published studies about compliance in the context of covid-19, these are survey studies. What our study adds is the opportunity to explore other influences on adherence/compliance e.g. structural aspects of the environment, economic concerns, cultural practices etc. within a rich description of experiences, beliefs and views.

That researcher that conducted the interviews and the Public Adviser who co-facilitated focus groups were from a Muslim background which help to facilitate discussion, facilitating participants to express universal terminologies used within the Muslim community.

We were only able to explore experiences of those people who wished to take part in the study and consequently our findings may not represent all sections of the Muslim community, being an opportunistic sample recruited through word of mouth, links to community leaders and social media. Despite a variety of routes to engage older community members we were unable to recruit participants older then 65, limiting our data directly from retired and older working community members, although respondents described their perceptions of older community members. We believe this barrier to research participation was at least partly due to language barriers within this age group, and a lack of familiarity with, or trust in, research as a process. Therefore, further research is important to capture the experiences of non-English speakers and older adults. Given that this study was conducted within the early period of the pandemic, there is potential for peoples’ experiences and perceptions of risk to have shifted over time.

## Conclusion

Perceptions of risk are influenced by lived experience of covid-19 and formal and informal guidance. The greater the perceived risk the more likely people were to seek to follow hygiene and distancing practices. Socioeconomic and structural factors influenced the extent to which people were able to, or felt able to, follow social distancing and isolation measures. Religious beliefs and a strong sense of responsibility were important drivers of behaviour, supporting key government messages about social distancing. Improved communication between local leaders and the provision of information in ways that are accessible to the BAME community are essential in delivering key public health messages.

## Supplementary Information

**Additional file 1.**

## Data Availability

The datasets generated and/or analysed during the current study are not publicly available as they may contain information that could compromise the confidentiality and anonymity of the participants but are available (limited) from the corresponding author on reasonable request.
