# Supplementary material for: How do Muslim community members perceive Covid-19 risk reduction recommendations - a UK qualitative study?
Source: BMC Public Health. 2021 Mar 5;21:449. doi: 10.1186/s12889-021-10506-4 (PMC7935005; doi:10.1186/s12889-021-10506-4)
Supplement: Supplementary file 1 — Additional file 1. [file 12889_2021_10506_MOESM1_ESM.docx]

**How do Muslim Community members perceive Covid-19 risk reduction recommendations - a UK qualitative study?**

**One to one Interview Topic Guide**

**Introduction**

To try to control the spread of the virus called COVID19, we are all being directed by UK Government to stay at home and only leave home for essential reasons including: travel to work/work – if we cannot work from home, to get essential food items and medications, to take exercise once a day or where we have specific caring responsibility for others outside of our own household [2m social distancing]. We are also being told to self-isolate – away from others if we are experiencing typical symptoms of the virus and also if other members of our household are experiencing symptoms.

We are seeking to understand better, what people know about COVID19 and how they think about and experience risk [e.g. of contracting the virus, risk and consequences of having the virus for themselves and their families]. We also want to understand more about peoples’ experiences of the current directives and restrictions - requiring us to socially distance and self-isolate from others.

**Understanding of COVID19/Coronavirus**

So perhaps first:

*Prompts*

- How would you describe what COVID19/Coronavirus is (understanding of what a coronavirus is)
- How do you think the virus is passed from one person to another (understanding of transmission)
- Where do you get information about COVID19/ coronavirus from?

**Risk**

What do you think the risks are to you from COVID19/ coronavirus?

*Additional prompts:*

- Risk of contracting the virus
  - If think at high risk – Why?
  - If think at less risk – Why?
  - Beliefs about risk (cultural and religious perceptions)
- Risk associated with the virus
  - Risks for self .e.g. hospitalisation, mortality, economic, emotional, psychological
  - Implications of hospitalisation e.g. religious/cultural/social
  - Risks for family e.g. same as for individual?

**Self-isolation**

Thinking now about the advice/directive to self-isolate if you were to have, or if you have had symptoms of the virus:

- What do you think of this advice on self-isolation?
- what has been your experience of trying to self-isolate? or family member

*Additional prompts:*

- Have there been any particular challenges to trying to self-isolation?
- How have those impacted your ability to self-isolate?
- What if anything has helped you to self-isolate?
- What has been your experience of self-isolation? e.g. loneliness, sadness, anxiety, low mood, practical difficulties – getting food, not being able to go to work/economic impact?

**Social/physical distancing**

Now, thinking about the current directive to stay at home and not meet with others from outside of your household:

- What do you think of this advice on social distancing?
- What impact has/is this having in your life currently?

*Additional prompts:*

- How easy or not has it been for you to follow the directive to stay at home/social distancing?
- Is there anything that has made it difficult for you to stay at home? [if not mention prompt about cultural/social/religious beliefs and expectations]
- What worries/concerns do you have about restrictions/ social distancing?
- What would make social distancing easier?

**Messages and directives**

- What do you think about the information that has been coming out from UK government/NHS England?
- How might this information be improved in the future to better inform people about risk?
- How might this information be improved in the future to help people comply with government directives?

Anything that I haven’t asked about that you would like to add before we end the interview?

Thank you for participating.

**Focus group Interview Topic Guide**

**Introduction**

To try to control the spread of the virus called COVID19, we are all being directed by UK Government to stay at home and only leave home for essential reasons including: travel to work/work – if we cannot work from home, to get essential food items and medications, to take exercise once a day or where we have specific caring responsibility for others outside of our own household [2m social distancing]. We are also being told to self-isolate – away from others if we are experiencing typical symptoms of the virus and also if other members of our household are experiencing symptoms.

As a group we are seeking to understand better, what people know about COVID19 and how they think about and experience risk [e.g. of contracting the virus, risk and consequences of having the virus for themselves, their families and the community]. We also want to understand more about peoples’ experiences of the current directives and restrictions - requiring us to socially distance and self-isolate from others.

**Understanding of COVID19/Coronavirus**

So perhaps first:

*Prompts*

- How would you describe what COVID19/Coronavirus is (understanding of what a coronavirus is)
- How do you think the virus is passed from one person to another (understanding of transmission)
- What do you think is the Muslim community’s understanding of COVID19?
- Where do you/community get information about COVID19/ coronavirus from?

**Risk**

What do you think the risks are to you from COVID19/ coronavirus?

*Additional prompts:*

- Risk of contracting the virus
  - If think at high risk – Why?
  - If think at less risk – Why?
- Beliefs about risk (cultural and religious perceptions)
- How do you feel about the Muslim community understanding and response to of risk? Are there any concerns that you have about the Muslim community understanding of risk
- Risk associated with the virus
  - Risks for self .e.g. hospitalisation, mortality, economic, emotional, psychological
  - Implications of hospitalisation e.g. religious/cultural/social
  - Risks for family e.g. same as for individual?

**Self-isolation**

Thinking now about the advice/directive to self-isolate if you were to have, or if you have had symptoms of the virus:

- What do you think of this advice on self-isolation?
- what has been your experience of trying to self-isolate? or family member

*Additional prompts:*

- Have there been any particular challenges to trying to self-isolate?
- How have those impacted your ability to self-isolate?
- What if anything has helped you to self-isolate?

**Social/physical distancing**

Now, thinking about the current directive to stay at home and not meet with others from outside of your household:

- What do you think of this advice on social distancing?
- What do you feel about the Muslim community thinking of this social distancing? E.g. closing of the mosques

*Additional prompts:*

- How easy or not has it been for you to follow the directive to stay at home/social distancing?
- Is there anything that has made it difficult for you to stay at home? [if not mention prompt about cultural/social/religious beliefs and expectations].
- How do you feel about this with the Month of Ramadan?
- What worries/concerns do you have about restrictions/ social distancing?
- What would make social distancing easier?

**Messages and directives**

- What do you think about the information that has been coming out from UK government/NHS England?
- How might this information be improved in the future to better inform people about risk?
- How might this information be improved in the future to help people comply with government directives?

Anything that I haven’t asked about that you would like to add before we end the interview?

Thank you for participating.
